# A new species of *Cilunculus* Loman, 1908 (Arthropoda: Pycnogonida: Ammotheidae) from the South-western Indian Ocean

**DOI:** 10.3897/BDJ.8.e49935

**Published:** 2020-03-17

**Authors:** Jianjia Wang, Dingyong Huang, Wentao Niu, Feng Zhang

**Affiliations:** 1 The Key Laboratory of Invertebrate Systematics and Application, College of Life Sciences, Hebei University, Baoding, China The Key Laboratory of Invertebrate Systematics and Application, College of Life Sciences, Hebei University Baoding China; 2 Third Institute of Oceanography, Ministry of Natural Resources, Xiamen, China Third Institute of Oceanography,Ministry of Natural Resources Xiamen China; 3 Fujian Provincial Station for Field Observation and Research of Island and Costal Zone in Zhangzhou, Zhangzhou, China Fujian Provincial Station for Field Observation and Research of Island and Costal Zone in Zhangzhou Zhangzhou China; 4 Fujian Provincial Key Laboratory of Marine Ecological Conservation and Restoration, Xiamen, China Fujian Provincial Key Laboratory of Marine Ecological Conservation and Restoration Xiamen China

## Abstract

**Background:**

A new species of *Cilunculus* was determined by the unique characteristic of the three distal processes present on the almost horizontal proboscis and the other differences, including a shorter and blind ocular tubercle, fewer setae on the legs and a glabrate trunk.

**New information:**

New *Cilunculus* species from the South-western Indian Ocean

## Introduction

The genus *Cilunculus* Loman, 1908 consists of 32 species (Bamber et al. 2018). Fry and Hedgpeth (1969) gave the diagnosis for this genus and described two species from the Antarctic. Stock (1955), Stock (1978), Stock (1997) provided keys to contemporary species and Nakamura and Child (1983) established a key to the Japanese species of *Cilunculus.* Five species were described since Stock’s key (Bamber et al. 2018) and Bamber (2013) had preliminarily discussed all these additional species including *Cilunculus misesetosus* Turpaeva, 2005 from the North Atlantic and other four species, *Cilunculus ateuchus* Bamber, 2004, *Cilunculus mergus* Bamber, 2004, *Cilunculus cymobostrychos* Bamber, 2004 and *Cilunculus roni* Bamber, 2013, from Melanesia. Bamber (2013) conjectured that *C. misesetosus* might be a junior synonym of *Cilunculus europaeus* Stock, 1978, but without providing any further study.

Several authors (Fry and Hedgpeth 1969, Pushkin 1973, Stock 1978) had recognised the close relationship between *Cilunculus* and *Ammothella* Verrill, 1900. However, the studies of Arango (2002), Nakamura et al. (2007) and Sabroux et al. (2017) made this outstanding problem more complicated, but up to now, still maintained the situation due to a lack of conculsive evidence one way or the other. Thus, the new species, described below, was still included under the genus *Cilunculus*.

Few specimens of Pycnogonida were obtained during the Chinese DY115-20 cruise which undertook the comprehensive survey on the hydrothermal vents along the Southwest Indian Ridge. Wang et al. (2013) had described one new species collected from station DY115-20VII-TVG02 which was close to the present station DY115-20VII-TVG04. Two specimens of *Cilunculus* were collected at this station and determined to be a new species.

## Materials and methods

The specimens were sorted from benthic fauna which were collected by washing the sediment obtained by a TV-grab from Station DY115-20VII-S04-TVG04 during the Chinese DY115-20 expedition on R/V Dayangyihao in February 2009. They were preserved in 90% ethanol at the Third Institute of Oceanography, Ministry of Natural Resources, China (Nos. 20VIIS4TVG04.01, 20VIIS4TVG04.02). The specimens were drawn using a Camera Lucida and photographs were taken with an Auto-montage system on a Leica M205 FA stereomicroscope. Measurements were made axially, dorsally for the trunk, laterally for the palp, proboscis and leg and are given in millimetres.

## Taxon treatments

### Cilunculus tricuspis

2020 sp. n.

D7217174-DF80-5113-8173-783DB2ED0575

urn:lsid:zoobank.org:act:C9065C82-03F0-4DDD-82D4-1C0B6A36DE68

#### Materials

- **Type status:** Holotype. **Occurrence:** catalogNumber: 20VIIS4TVG04.01; individualCount: 1; sex: male; lifeStage: adult; **Taxon:** kingdom: Animalia; phylum: Arthropoda; class: Pycnogonida; order: Pantopoda; family: Ammotheidae; genus: Cilunculus; specificEpithet: tricuspis; **Location:** locationID: South-western Indian Ocean; verbatimDepth: 1585 m; verbatimLatitude: 37.466S; verbatimLongitude: 51.729E; decimalLatitude: -37.466; decimalLongitude: 51.729; **Identification:** identifiedBy: Jianjia Wang, Dingyong Huang, Wentao Niu, Feng Zhang; **Event:** samplingProtocol: TV-grab; year: 2009; month: February; day: 7; **Record Level:** institutionID: Third Institute of Oceanography, Ministry of Natural Resources; institutionCode: MNRTIO
- **Type status:** Paratype. **Occurrence:** catalogNumber: 20VIIS4TVG04.02; individualCount: 1; sex: male; lifeStage: adult; **Taxon:** kingdom: Animalia; phylum: Arthropoda; class: Pycnogonida; order: Pantopoda; family: Ammotheidae; genus: Cilunculus; specificEpithet: tricuspis; **Location:** locationID: South-western Indian Ocean; verbatimDepth: 1585 m; verbatimLatitude: 37.466S; verbatimLongitude: 51.729E; decimalLatitude: -37.466; decimalLongitude: 51.729; **Identification:** identifiedBy: Jianjia Wang, Dingyong Huang, Wentao Niu, Feng Zhang; **Event:** samplingProtocol: TV-grab; year: 2009; month: February; day: 7; **Record Level:** institutionID: Third Institute of Oceanography, Ministry of Natural Resources; institutionCode: MNRTIO

#### Description

**Figure 1.**
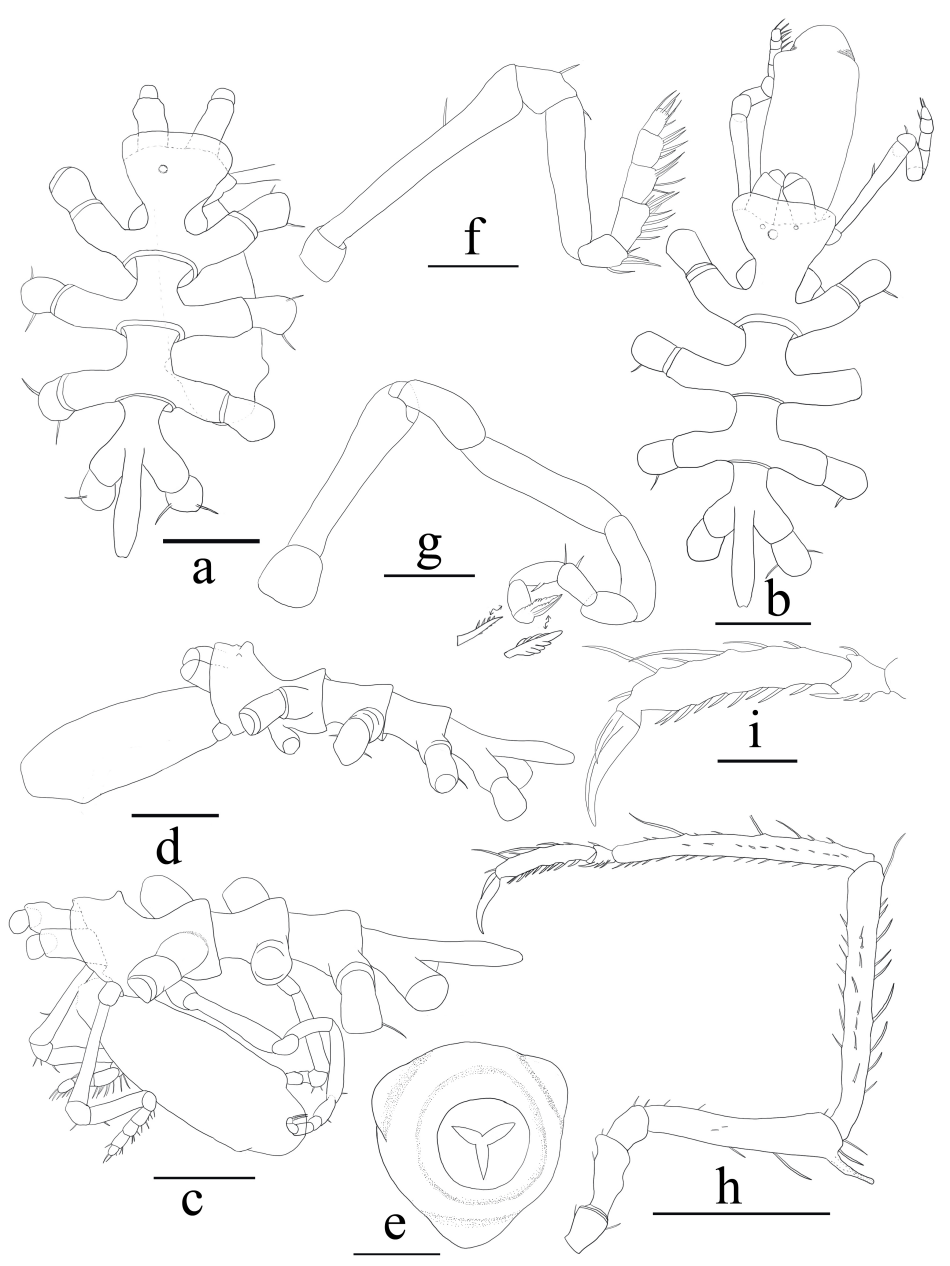
*Cilunculus tricuspis* sp. n., male; holotype: **a.** trunk, dorsal view; **c.** trunk, lateral view; **e.** proboscis, frontal view; **f.** palp; **g.** oviger; **h.** leg 3; **i.** tarsus, propodus and claws of leg 3, enlarged. Male; paratype: **b.** trunk, dorsal view; **d**. trunk, lateral view. Scale bars a-d = 0.5 mm; e, f, g, i = 0.2 mm; h = 1 mm.

Body length 1.79 mm. Trunk glabrous (Fig. 1a-e), completely segmented, dorsal segmentation lines raised and swollen. Lateral processes slender and smooth, well separated by a little more than their own diameter. Cephalon extends anteriorly into a hood, over 1/5 of the length of the chelifores. Ocular tubercle inconspicuous, dome-shaped, without eyes. Abdomen spindle-shape, not articulating to the body, extends horizontally to the middle of the second coxae of leg pair 4.

Proboscis barrel-shaped, 0.7 times as long as trunk, with two dorsal and one ventral triangular processes close to mouth.

Chelifores stout, with one-articled scape; chela atrophied, without fingers.

Palp of nine articles (Fig. 1f); article 2 longest, almost 1.5 times as long as article 4; distal five articles very short, each with row of ventral setae.

Oviger glabrous (Fig. 1g); article 2 longest, slightly longer than article 4; distal five articles short, of decreasing length, article 7 to article 10 with compound spines, formula 1:1:1:2, spines on article 10 thicker.

Third leg (h-i) slender, longest articles with long setae. First coxa short, with few setae; second coxa 1.5 times as long as first or third coxa, with ventrodistal and dorsal protuberances; femur 2.6 times as long as second coxa, with dorsal and distal long setae, tall cement gland tube dorso-distally; first tibia slightly longer than second tibia and 1.4 times longer than femur, with dorsal, lateral and ventral rows of setae; second tibia with ventral and lateral rows of setae and sparse dorsal setae; tarsus small, subtriangular, with one protuberance dorsally, one spine and few setae ventrally; propodus without heel, sole with seven spines and two distal setae, with two long setae dorsally and some short setae dorsally and distally; main claw slender, gently curved, 0.6 times as long as propodus; auxiliary claws half the length of main claw.

Female unknown.

*Measurements of holotype in mm*: trunk length from the anterior margin of the cephalon to the tip of 4th lateral processes 1.79; width across second lateral processes 1.0; proboscis length 1.27; abdomen length 0.55. Chelifore scape length 0.24. Palp article 1 (Pa1) 0.07; 2 (Pa2) 0.51; Pa3 0.08; Pa4 0.33; Pa5 0.08; Pa6 0.09; Pa7 0.08; Pa8 0.07; Pa9 0.06. Oviger article 1 (O1) 0.11; O2 0.39; O3 0.16; O4 0.35; O5 0.21; O6 0.10; O7 0.08; O8 0.08; O9 0.07; O10 0.02. Third leg: coxa-1 0.26, coxa-2 0.40, coxa-3 0.24, femur 1.06, tibia-1 1.49, tibia-2 1.41, tarsus 0.11, propodus 0.54, main claw 0.32, auxiliary claw 0.16.

#### Etymology

This specific name is from the Latin *tricuspis* (three-pointed), referring to the three processes on the proboscis.

#### Distribution

This new species was found only at the type locality, the substrate of which consisted of white and yellow foraminiferan oozing along with dead coral and shells and a small amount of black basalt.

#### Taxon discussion

According to the key given by Stock (1997), this new species would identify as *Cilunculus haradai* Nakamura & Child, 1983 and the common characteristics between these two species impelled us to consider this new species as a deep-sea form of *C. haradai*. After further examination, several differences with *C. haradai* convinced us to establish this new species, as it presented shorter and blind ocular tubercle, glabrous lateral processes, fewer setae on legs, absence of heel spines and almost horizontal proboscis with three distal processes.

*Cilunculus tricuspis* n. sp., keying to couplet 15 of Stock’s key, was distinct from *C. cymobostrychos* and *C. roni* in glabrous trunk, without wavy barbed setae or tubercles and distinguished from *C. mergus* and *C. roni* by absence of a dorsal hump on the propodus and is also different from *C. misesetosus* which has long auxiliary claws almost equal to the main claw.

Amongst the 32 species, only *Cilunculus australiensis* Clark, 1963, *Cilunculus galeritus* Nakamura & Child, 1991 and *C. roni* showed the proboscis adorned with processes, but the new species could be easily distinguished from *C. australiensis* which presented tall spinose tubercles on the trunk and *C. galeritus* which presented the unique larger cephalic segment hood and could be distinguished from *C. roni* by the tubercles on the trunk and legs.

**Figure 2.**
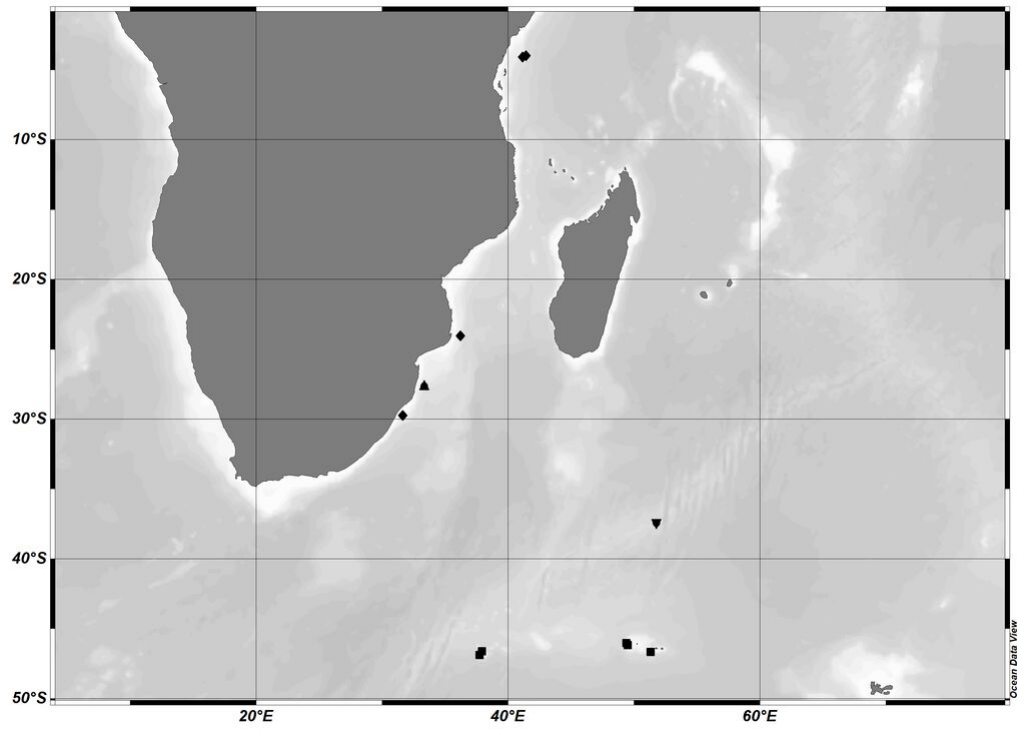
The collecting station for station of *Cilunculus tricuspis* n. sp. and the distribution of four species of *Cilunculus* ▼-*C. tricuspis* n. sp.; ▲-*C. bifidus*; ■-*C. kravcovi*; ◆-*C. sewelli*.

There are few species of *Cilunculus* reported from the Indian Ocean. Amongst these are *Cilunculus bifidus* (Stock, 1968) only found off False Bay (South Africa) (1361 m) (Stock 1968), *Cilunculus kravcovi* Pushkin, 1973 which is found in the Crozet Islands (255-309 m) and the Prince Edward Islands (South Indian Ocean) (360-376 m) (Pushkin 1973, Arnaud and Branch 1991) and *Cilunculus sewelli* Calman, 1938 which was found off Zanzibar (1789 m), Natal (South Africa) (440 m), Kenya (1520 m) and the Mozambique Channel (1628-1600 m) (Calman 1938, Fage 1956, Stock 1968). The new species was relatively close to *C. kravcovi* (Crozet Islands), although they occur several hundred kilometres apart (Fig. 2). The three recorded species were differentiated from *C. tricuspis* n. sp. based on their distinctive characteristics, such as *C. bifidus* having a tall and bifid ocular tubercle, *C. kravcovi* and *C. sewelli* having an acute ocular tubercle and noticeably long setae on legs.

## Supplementary Material

XML Treatment for Cilunculus tricuspis
